# Patterns of Reimperialization and Postimperial strategic cultures. The cases of Liberal Spain (1833-1868) and Post-Soviet Russia (1991-2025)

**DOI:** 10.12688/openreseurope.21137.1

**Published:** 2025-09-02

**Authors:** Rodrigo Escribano Roca

**Affiliations:** 1 Department of Global and International History, Institute of History, Consejo Superior de Investigaciones Cientificas, Madrid, Community of Madrid, 28802, Spain; 2 Center of American Studies, Universidad Adolfo Ibanez, Santiago de Chile, Región Metropolitana, 2520000, Chile

**Keywords:** Reimperialization; Post-imperial conflicts; Post-Soviet world; Latin America; Comparative historical sociology.

## Abstract

This article explores the structural patterns of post-imperial conflict through a comparative analysis of Liberal Spain (1833–1868) and post-Soviet Russia (1991–2025). It argues that both powers, following the abrupt collapse of their empires, developed strategic cultures oriented toward reimperialization—defined as the attempt to reassert influence over former imperial domains through cultural, diplomatic, or military means. Drawing on theories from historical sociology, international relations, and strategic studies, the article introduces the concepts of “levels of imperiality” and “degrees of rupture” to explain why both Spain and Russia failed to construct stable post-imperial states and instead pursued increasingly coercive strategies to restore lost power. The study identifies a recurring six-stage pattern: (1) imperial collapse, (2) internal disintegration, (3) soft reimperialization through diplomacy and cultural outreach, (4) frustration and strategic shift, (5) militarized reimperialization, and (6) confrontation with geopolitical realities. This model is applied to the Spanish intervention in South America (1864–1866) and the Russian invasion of Ukraine (2022–present), revealing striking parallels in their strategic rationales, ideological justifications, and operational outcomes. The article concludes that post-imperial reimperialization is more likely when imperial collapse is abrupt, when successor states are internally divided, and when the international system is permissive. By revisiting two cases often treated as peripheral in the historiography of decolonization, the article offers a long-term framework for understanding the persistence of imperial logics in global politics and the strategic cultures that sustain them.

## Plan language summary

This article compares two very different moments in history to understand how former empires try to regain their lost influence. It looks at Spain in the mid-1800s, after it lost most of its American territories, and Russia today, after the breakup of the Soviet Union. The study shows that both countries went through similar stages: first they collapsed, then they tried to rebuild peacefully, and finally they turned to military force to try to reassert control over former territories. Spain’s intervention in South America in the 1860s and Russia’s invasion of Ukraine are used as examples. Although separated by time, technology, and geography, both cases reveal how certain political cultures—shaped by past imperial power—can lead countries to repeat patterns of aggressive behavior. The article helps explain why some post-imperial powers struggle to move on, and why they may choose war to try to restore their former status in the world.

## 1. Introduction

The Hispano-South American War ^1^ was a conflict between the Spanish Monarchy and the republics of Chile, Peru, Ecuador, and Bolivia. The hostilities primarily unfolded at sea, consisting of minor naval engagements, blockades, and the bombardments of the ports of Valparaíso and Callao by the Spanish Royal Navy ^2^. These events occurred between September 1865 and May 1866, although the conflict remained frozen until an armistice was signed in 1871. The immediate cause of the war was the capture of the Chincha Islands in April 1864 by the Spanish fleet under Admiral Luis Hernández Pinzón. Though small, these islands were the Republic of Peru's leading source of guano, a key export on which its treasury relied ^3^. Spain sought to coerce its former colony into signing an unequal treaty granting commercial and diplomatic privileges ^4^. The Ministry of State and naval officers assumed the operation would be swift and low-cost. However, Peru’s resistance, supported by its South American neighbors, transformed the intervention into a protracted and costly war for the former metropole ^5^.

This article compares the Hispano–South American War (1865–1871) and the Russo-Ukrainian War (2022–present), focusing on the dynamics that led to their outbreak. We argue that both wars were triggered by unresolved imperial legacies. In each case, the conflict was incubated during a critical juncture in which Spain (1833–1864) and Russia (1991–2022), having lost substantial portions of their territorial empires, developed strategic cultures inclined toward reasserting influence over their former imperial peripheries. As a result, both evolved into post-imperial revisionist states pursuing reimperialization. We define “reimperialization” as a process through which certain actors try to restore imperial institutions or impose some control of the old metropole over the territorial domains formerly held by an empire ^6^.

We aim to use this comparative approach to identify historical and strategic patterns that might inform an interdisciplinary theory of post-imperial conflicts. This category refers to interventions or wars resulting from geopolitical tensions that follow the sudden collapse of an empire. Although several conflicts arguably fit this typology —such as the Anglo-American War (1812) ^7^, the Balkan Wars (1912–1913) ^8^, or French interventions in Africa (1960–2014) ^9^— no systematic theorization exists within strategic studies, international relations theory, or historical sociology.

Such theoretical exercise is increasingly necessary. In a recent work, Jeffrey Mankoff argues that today's revisionist powers challenging the liberal international order are, without exception, post-imperial states undergoing processes of reimperialization ^10^. Russia, China, Turkey, and Iran all fit this type. These political entities were once the cores of vast empires. They now claim the right to exert cultural, political, and economic hegemony over the "successor states" that emerged from their dissolution. They all clash with Westphalian and Wilsonian doctrines enshrining sovereign equality, non-intervention, and national self-determination. From their perspective, these doctrines are legal fictions that have enabled U.S. dominance over the international system. In contrast, their governments propose a world organized into spheres of influence, aiming to restore their former imperial domains as far as possible. The war in Ukraine, the escalation in the Middle East, and territorial disputes in the South China Sea can all be interpreted through this analytical lens ^11^.

This raises key questions: Are there long-term historical patterns that explain post-imperial irredentism? Why do some decolonization processes yield peaceful transitions and stable post-imperial sovereignties, while others lead to failed states and chronic conflict? In recent decades, scholars have explored imperial legacies extensively, particularly regarding cultural structures and the shaping of international legal norms ^12^. However, as Julian Go ^13^ has recently noted, these lines of research often fall short in identifying the specific ways post-imperial dynamics shape global geopolitics.

This article seeks to identify patterns and sequences that might define the structural features of post-imperial conflicts, focusing on their strategic, institutional, and political-diplomatic dimensions. The choice of cases may seem unexpected. The historical scenario in which Spain bombarded the main ports of Chile and Peru bears little resemblance to Russia's missile attacks on eastern Ukrainian cities. Spain acted with a modest steam-powered fleet isolated from the Iberian Peninsula. Putin's Russia employs hypersonic missiles and hundreds of thousands of well-equipped troops deployed along contiguous borders. The international contexts of 1865 and 2025 appear radically different. The chronological, technological, and geopolitical gaps are stark.

Yet we selected these cases for two key reasons. First, as we will demonstrate in subsequent sections, the reimperialization processes pursued by Isabeline Spain (1833–1868) and Post-soviet Russia (1991-present) followed comparable historical sequences. Both transitioned from imperial collapse to failed attempts at peaceful post-imperial transition, and ultimately to aggressive strategies of reimperialization that culminated in unintended wars. As Steinmetz suggests, this dialectic between spatial-temporal difference and processual similarity is ideal for meaningful historical comparison ^14^. Second, such comparison enables a long-term perspective that challenges the linear narrative that has shaped much of the historiography on decolonization. The mainstream focus on the post-World War II wave of decolonization in Asia and Africa has often relegated the Spanish and Soviet experiences to mere prologue or epilogue ^15^. Yet both deviate from the classic paradigm in which colonial empires give way to stable nation-states. As we will show, the Spanish and Russian cases are better understood as post-imperial laboratories marked by unresolved imperial continuities at both domestic and international levels ^16^. The contrasted examination of these experiences will likely enrich the time horizons that mark strategic planning in post-imperial spaces ^17^. These horizons are conditioned by a teleological perspective of the processes of decolonization, which assumes as a historical law the universalization of the sovereign nation-state that was typical only in the second half of the twentieth century ^18^.

## 2. Levels of imperiality, institutions and strategic cultures: a theoretical framework

Our methodology draws on historical sociology, particularly the theories of serial contextualism ^19^ and structural studies of imperial cycles ^20^. The third section offers a comparative and analytical historical narrative of the sequences that led to Spain's and Russia's reimperializations. These are grounded in current historiography and our own research. The fourth section will combine insights from strategic culture studies, historical institutionalism, and comparative historical sociology to identify the key variables connecting both cases. This final segment aims to take a first step toward building an interdisciplinary theory of post-imperial conflict.

Before proceeding to the historical analysis, defining some key concepts drawn from the disciplinary fields referenced above is necessary. First, we are interested in determining the “levels of imperiality” of Isabelline Spain and post-Soviet Russia. Although the expression is a neologism, it is inspired by institutionalist studies of imperial legacies —such as those by Daron and Acemoglu ^21^ and James Mahoney ^22^— which examine their influence on former dependencies by appealing to the notion of “levels of colonialism”. We deliberately avoid terms such as colonial and postcolonial since “colony” generally refers to a specific type of imperial relationship based on settlement and plantation ^23^. The concepts of “postimperial” and “levels of imperiality” offer more significant theoretical and comparative potential and allow us to evaluate the experiences of metropolitan centers such as Spain and Russia. The term “imperiality”, recently adopted by Byzantine scholars Blanchet and Estangui ^24^, derives from Roland Barthes’ conceptualization, which denoted the role played by the mythification of an imperial past in shaping the identity and politics of a postimperial society ^25^. We seek to expand the concept by combining it with institutionalist notions. When we speak of “levels of imperiality”, we refer to the constellation of institutional, ideological, and socioeconomic structures that an imperial system bequeaths to a postimperial state or society—and the relative weight these structures carry in comparison with those emerging from processes of de-imperialization ^26^.

In this regard, it is also worth recovering the concept of “degrees of rupture”, which was used in the outstanding comparative work of Cummings and Hinnebusch on the postimperial trajectories of the Middle East and the Caucasus. They argue that it is essential to assess the extent to which the collapse of an imperial system gives rise to political cultures that seek to repudiate the institutions built around that system and to what degree these cultures succeed in establishing new frameworks of power that break away from imperiality ^27^. Attention to the levels of imperiality and degrees of rupture that conditioned the postimperial experiences of Spain and Russia will be essential for understanding the complex temporal dialectic between continuity and change that underpins their re-imperializing trajectories.

To this end, we will focus on the evolution of postimperial institutions and their strategic cultures. Drawing on sociological and historical institutionalism theories, we define an “institution” as a generalizable set of rules and procedures that guide the behavior of its members and promote predictable patterns of interaction—whether consensual or conflictual. These rules and procedures take the form of ideas, narratives, and schemas internalized by individuals as knowledge ^28^. The concept of “strategic culture” is closely related to this. In this work, we understand a strategic culture, following the institutionalist perspective proposed by Libel, as the institutionalized set of habits of thought, ideas, emotional responses, and behavioral patterns that define an epistemic community’s position regarding the uses of hard and soft power by a polity in international affairs ^29^. One could argue that strategic culture is a particularly relevant institution for defining foreign and military policy.

According to historical institutionalism, institutions generate trajectories of path dependence —that is, they tend to induce their members to reproduce their prescriptions over time, thus generating continuity ^30^. A strategic culture, consequently, aspires to reproduce itself over the long term, as a set of ideas and practices transmitted intergenerationally that seek to prescribe the geopolitical behavior of a state ^31^. However, change and transformation also disrupt these temporal patterns. To account for this, historical institutionalism has employed the category of “critical junctures”—periods or phases in which a confluence of external experiences or internal disputes leads to the alteration of institutional codes or even the disappearance of the institution itself ^32^. From this, it follows that a power’s strategic culture can be altered by critical junctures arising from significant historical experiences —whether in domestic or foreign policy— as well as by events occurring within or beyond its borders or through dialectical engagement with other strategic cultures or subcultures ^33^.

The cycles of persistence and rupture that shape an institution’s or strategic culture’s trajectories can be conceptualized as “sequences”. In this article, we argue that the structural repetition of the rhythms and dynamics of such sequences constitutes historical patterns ^34^. Here, emulating Go’s work on imperial expansion and contraction processes ^35^, we seek to identify the patterns that governed the postimperial strategic cultures of Russia and Spain, establishing parallels between their respective sequences of imperial crisis, transition, and re-imperialization. Of course, these dynamics were intimately linked to the constraints imposed by the international system. The comparative evaluation of the strategic cultures of both powers—and of the institutional and socioeconomic structures that shaped them —will allow us to develop an explanation that combines constructivist and realist approaches insofar as our analysis integrates both ideological factors and systemic constraints ^36^, enriching the argument with tools from the comparative sociology of empires ^37^.

The following sections will outline a narrative aimed at identifying the defining features of the postimperial strategic cultures of Spain and Russia: how they became institutionalized, what political subcultures composed them, what path dependence dynamics sustained them, which critical junctures transformed them, and what sequential patterns ultimately led them toward re-imperialization.

## 3. Patterns of crisis, transition and reimperialization

### 3.1. Imperial collapses and internal disintegrations

The Spanish Empire, whose territorial integrity was reaching its end at the dawn of the nineteenth century, was not a mere sum of a metropolitan state and its dependencies. Instead, it could be more accurately defined as a composite or polycentric imperial monarchy ^38^. Built over three centuries, it consisted of a vast jurisdictional network of kingdoms, municipalities, and corporations stretching across the Iberian Peninsula, the Americas, and the Philippines ^39^. This framework was held together not mainly by modern state centralization but by pillars characteristic of the political culture of the Ancien Régime: loyalty to the Crown, the Catholic religion, the Spanish language, and a diffuse sense of Hispanic identity, particularly vivid among the peninsular and American elites ^40^. More than a state with colonies, it was an intercontinental conglomerate of powers governed by legal pluralism, local autonomy, and a strict corporate hierarchy.

The balance of this institutional structure began to falter with the Bourbon reforms, which—aiming to expand the Crown’s fiscal and administrative prerogatives to compete with France and Britain—strained the imperial pact that had historically granted Spanish American elites a high degree of autonomy ^41^. The Napoleonic Wars (1803–1815) and the cycle of liberal revolutions that accompanied them (1808–1823) led to a power vacuum both in Spain and Spanish America ^42^. The empire collapsed suddenly due to the explosive convergence of long-term disintegrative dynamics—tensions between centralization and autonomy, the inability of the corporate monopoly system to harmonize economic development and social power structure—and the external shock represented by the upheavals and revolutions of the period ^43^. 

The French occupation of nearly the entire Iberian Peninsula cast doubt on the viability of the imperial system. In a desperate attempt to preserve the empire’s integrity, the Cortes convened in Cádiz in 1810 proposed an innovative formula: to refound the monarchy as a globally scaled representative national state. To this end, they invited Spanish American representatives to participate in drafting the 1812 Constitution, aiming to grant them citizenship and parliamentary representation in a shared political project. Nevertheless, the experiment failed ^44^. The American deputies’ demands for proportional representation and commercial liberalization clashed with opposition from peninsular mercantile lobbies and a racialized, centralist vision of empire held by much of the Spanish liberal establishment ^45^. Ultimately, the strategic culture institutionalized by the Bourbon state in the previous century prevailed. This culture was grounded in the belief that maintaining the empire —with its commercial privileges, patronage, and tax resources— was necessary to ensure the monarchy’s prosperity and security ^46^. The architects of the Bourbon reforms assumed that imposing the prerogatives of the Crown and peninsular administrations over American local powers was essential to achieving those goals ^47^. This strategic culture gained institutional embodiment under Bourbon reformism and appears to have entered a path-dependent dynamic that explains the refusal of both Spanish liberals and absolutists to grant the Americas political autonomy or equal representation ^48^. This inertia blocked reform and accelerated the transition of American autonomist movements into full-fledged independence efforts, further exacerbated by Spain’s growing political instability following the return of Ferdinand VII.

Madrid’s refusal to negotiate autonomist reforms or controlled emancipations with the American elites—which might have secured at least some preferential diplomatic or commercial arrangements—shaped the course of the conflict ^49^. Even after the definitive royalist defeat at Ayacucho (1824), Ferdinand VII’s absolutist administrations concocted illusory reconquest projects ^50^. It was only when the liberals returned to power in 1833, amid political, economic, and military desperation, that formal recognition of the Spanish American independencies became feasible. On December 1, 1836, the Cortes solemnly acknowledged that the monarchy had lost its former “colonies.” However, the statement was misleading: Spain had not merely lost a few peripheral dependencies; it had lost itself ^51^. 

Indeed, the contrast between the Catholic Monarchy of 1803 and the liberal Spain of 1836 was stark. The post-emancipation state was territorially and demographically shrunken—with 12 million fewer square kilometers and 12 million fewer subjects—economically devastated—with a 40% collapse in exports, once based on imperial monopolies—and fiscally anemic, having lost the vital revenue streams generated by the viceroyalties, particularly New Spain ^52^. The Spanish Royal Navy, once a pillar of imperial power, had dwindled from over 200 major ships to barely a dozen operable vessels by 1834. This military decline mirrored the country’s geostrategic bankruptcy ^53^. 

The material collapse was compounded by internal political decomposition. The tension-filled coexistence of jurisdictional monarchy and Bourbon centralization attempts during the eighteenth century had at least provided a semblance of legal-political order. The irruption of liberal constitutionalism tore through the old system’s seams. The Peninsula spiralled into polarization between democratizing projects, absolutist nostalgia, and violent reactions ^54^. Disagreements between moderate and progressive liberals paralyzed any effort to consolidate a new order. The country became trapped in a cycle of pronunciamientos, ministerial instability, and legitimacy crises ^55^. 

To make matters worse, what remained of the monarchy was engulfed in civil war. After Ferdinand VII died in 1833, liberal support for the regency of María Cristina and the succession of Isabel II was challenged by Carlism, which rallied anti-liberal and regionalist factions behind Infante Don Carlos. This uprising was not merely a dynastic dispute but the crystallization of an alternative political project aimed at refounding the monarchy on traditionalist principles ^56^. With a court established in Estella and substantial territorial control in the Basque Country, Navarre, Catalonia, and the Maestrazgo, the Carlists effectively split Spain into two competing states—each receiving support from different European powers, with liberal Spain enjoying the backing of pro-constitutional France and Britain ^57^. The civil war of 1833–1840 revealed the impossibility of forging a new national articulation following the collapse of the imperial order. The wars of independence that had fractured the empire now continued on the Peninsula, itself operating as a dysfunctional fragment of empire.

In this context, the recognition of Spanish American independence was less an act of reconciliation than a confession of defeat. Spain, in its prostration, had no other option. Not even its remaining possessions—Cuba, Puerto Rico, and the Philippines—provided comfort, as their status within the new liberal order remained contested. Should they be integrated as equal provinces or treated as external colonies? ^58^ The question revealed a more profound identity crisis. For many Spanish politicians and intellectuals, the only viable way forward was to reinvent the Hispanic community on new cultural, diplomatic, and commercial foundations. Liberal thinkers saw pan-Hispanism as the only path to saving the monarchy from economic decline and internal disintegration ^59^.

This post-imperial state of confusion did not constitute a historical anomaly. It is comparable to the collapse of the Soviet Union in 1991. Like the Spanish Monarchy, the USSR did not undergo an orderly imperial dismantling that preserved the stability of its core. Constitutionally, the Soviet Union had been structured as a federation of fifteen autonomous republics, held together less by legal cohesion than by the power network of the Communist Party and the binding force of communist ideology ^60^. As with Spain, the disintegration of the Soviet empire stemmed from a combination of long-term and short-term factors. The inability of state socialism to compete with Western economies, rising discontent with the political authoritarianism of a gerontocratic elite, and symptoms of imperial overstretch —evident in the failed intervention in Afghanistan and the mounting costs of sustaining satellite regimes in Eastern Europe since the late 1960s— all played a role ^61^.

The political and economic reforms initiated by Gorbachev sought to modernize the USSR and preserve its unity by reshaping its institutional framework. Democratization, liberalization, and renewed political-military cohesion were conceived as the new pillars of legitimacy. However, the reforms unleashed a power struggle between the conservative sectors of the CPSU and the emerging leaderships in the republics. ^62^ What began as internal tensions evolved into a confrontation between the central Soviet apparatus and the increasingly autonomous republican elites. Although secessionism had been a marginal option —except in the Baltic republics— the polarization of 1990, coupled with a deepening economic crisis, triggered the unanticipated disintegration of the Union. The attempt to forge a new mechanism of imperial unity through the proposed Union of Sovereign States failed amidst this power struggle, culminating in the failed coup d'état by conservative elements of the CPSU in August 1991 ^63^.

The Soviet Union and the Russian Federation that emerged from its collapse differed profoundly. A review of fundamental indicators highlights the magnitude of this rupture. The USSR occupied a territory of 22.4 million square kilometers. Russia was left with only 17.1 million following the independence of former Soviet republics such as Armenia, Azerbaijan, Belarus, Estonia, Georgia, Kazakhstan, Kyrgyzstan, Latvia, Lithuania, Moldova, Tajikistan, Turkmenistan, Uzbekistan, and Ukraine ^64^. The economic picture was no better. The final two decades of the twentieth century marked the full onset of Soviet economic decline. Between 1980 and 1999, the Russian GDP shrank by 50%. By the close of the century, the economy was paralyzed between an oversized informal sector, residual features of state socialism, and an oligarchic model of hydrocarbon-driven capitalism ^65^. Demographic trends also reflected this decay. The USSR had a population of 290 million in 1991; Russia inherited only 148 million—a demographic loss roughly equivalent, in proportional terms, to Spain’s after the collapse of the Catholic Monarchy. Worse still, the socio-economic dynamics of the 1990s unleashed a declining trend that projects the Russian population to fall below 120 million by 2050 ^66^.

Under such conditions, it is unsurprising that the USSR’s status as a superpower was not inherited by the Russian Federation, at least not in the 1990s. The military decline was not as drastic, in relative terms, as Spain’s after losing its viceroyalties: Russia retained a formidable nuclear arsenal and significant conventional forces. However, throughout the 1990s, these were undermined by chronic underinvestment, obsolete equipment, and an inability to project force beyond regional theaters ^67^.

As in Spain’s case, the military and economic shortcomings of the new order had clear political ramifications. Just as Spain was torn between Carlists and liberals, post-imperial Russia experienced the continuation of the same centrifugal tendencies that had unravelled the Soviet Union. The new Russian Federation was, in essence, a federal state composed of districts, territories, regions, cities, and republics with varying degrees of autonomy. While President of the Russian Federation, Yeltsin had encouraged the decentralization of the USSR to weaken Gorbachev’s power. After the Union’s collapse in 1991, however, this strategy backfired. Various Russian territories began demanding more autonomy or even independence. As head of state, Yeltsin attempted to reverse course and reassert control over Moscow through erratic and authoritarian measures, such as the military dissolution of parliament in 1993 and the imposition of a hyper-centralized constitution ^68^.

Eventually, the instability generated by this policy acquired an ethnic dimension. Although Russia remained ethnically homogeneous (with roughly 80% of its population identifying as Russian), pockets of resistance threatened renewed fragmentation. The most emblematic case was Chechnya, whose regional parliament declared independence in the fall of 1991. The republic functioned as a de facto independent state for three years until Yeltsin launched a military intervention that would last until 1996 ^69^.

Although the Chechen Wars and the Carlist Wars were radically different, both emerged as post-imperial conflicts. The collapse of the Catholic Monarchy and the USSR was followed by the disintegration of their political cores and the implosion of their legitimizing ideologies. In both cases, civil war became the primary mechanism for redefining power distribution in the post-imperial landscape. Thus, the Carlist and Chechen Wars were civil conflagrations deeply embedded in the broader processes of imperial dismemberment. Each catalyzed its respective national decline. Finally, just as the ideological foundations of the Spanish Monarchy crumbled alongside its empire, the Soviet ideological apparatus disintegrated simultaneously with its imperial structure.

The search for a new basis of legitimacy in post-Soviet Russia hinged on two elements. On the one hand, the promise of capitalist modernization was quickly replaced by a system marked by inequality, inefficiency, and extractive oligopolies—particularly in the hydrocarbon sector ^70^. On the other, Yeltsin’s Russia attempted to deploy nationalism as an identity glue. However, the centrifugal dynamics of the 1990s, compounded by ideological clashes among statists, liberals, Slavophiles, Westernizers, and Orthodox factions, prevented the articulation of a unifying national discourse. The resulting Russian Federation was an oligarchic and militarized polity governed by clientelist networks and extra-institutional codes of power ^71^.

In light of this analysis, we can unequivocally state the central claim of this section: the disintegration of the Spanish Monarchy and the Soviet Union was not, strictly speaking, a decolonization process that left the metropolitan core intact. Instead, they were sudden and profound implosions that directly affected the imperial centers, destroying them demographically, economically, and politically. Both imperial collapses resulted in disarticulating and deinstitutionalising their metropolitan political systems and legitimizing ideologies, temporarily turning them into failed states. The post-imperial strategic cultures that later emerged in Spain and Russia were rooted in these foundational breakdowns, and in the perception of various elite groups that there would only be corrected by recovering the path to empire.

### 3.2. Frustrated postimperial transitions

The collapse of the Spanish and Soviet empires did not, therefore, entail a complete acceptance of loss or renunciation of influence over their former domains. On the contrary, Spain and Russia attempted to articulate cultural and diplomatic hegemony projects that would enable them to retain residual authority over the territories that had once formed part of their imperial communities. The severe consequences of imperial collapse in both countries ultimately led to the institutionalization of strategic cultures that linked internal regeneration to the creation of post-imperial spheres of influence. In both cases, these aspirations initially took the form of peaceful and voluntary integration proposals. Spanish pan-Hispanism in the 1830s and post-Soviet internationalism in the 1990s shared the expectation that historical, linguistic, legal, and economic ties could create new forms of post-imperial unity. However, this idealistic phase soon led to a period marked by disappointment, imperial nationalism, and the temptation of interventionism.

In the Spanish case, following the formal recognition of independence in 1836, the liberal government embraced the idea of reconstructing a Hispanic community based on international law and commercial cooperation. The hope was that cultural affinities and the institutional legacy of the Catholic Monarchy would create the conditions for an alliance of Spanish-speaking nations led by Spain. Through treaties of friendship and commerce, the new Spanish constitutional State aimed to recover part of its lost influence and economic exchanges while simultaneously stabilizing the parliamentary order within the Peninsula ^72^. It was a diplomacy of cultural continuity, grounded in the belief that a shared language, a common religion, and a certain nostalgia for the viceregal order—fueled by the civil strife and public disorder afflicting the new republics—would act as catalysts for reconciliation ^73^.

This vision materialized into a concrete policy: the recognition of the new American states was accompanied by the expectation that they would assume part of the viceregal debt, return properties confiscated during the wars of independence, and grant commercial privileges to the former metropole ^74^. From the perspective of Spanish liberals, particularly the Progressives, a network of treaties would facilitate Spain’s reintegration into the Atlantic trade circuit and, consequently, its internal regeneration. The new Hispanic community was envisioned as liberal, constitutional, and cooperative. This ideal was not meant to challenge the hegemony of the great powers—especially Britain and, to a lesser extent, France—but rather to complement it. The objective was not for liberal Spain to displace these powers in the Hispanic Atlantic but to join them in projecting European commerce in the Americas ^75^.

However, this illusion quickly faded. The treaties were negotiated slowly, faced numerous diplomatic obstacles, and, when signed, proved disappointing ^76^. Several states, such as Peru and Colombia, openly rejected the idea of assuming the viceregal debt and were reluctant to grant tariff concessions ^77^. In other cases, such as Mexico or Venezuela, preferential agreements had already been signed with powers like Britain or France, placing Spain at a clear competitive disadvantage. Even where more favorable treaties were concluded—as in Ecuador or Bolivia—the benefits were meager and failed to meet the strategic ambitions of the Madrid government ^78^.

Spanish diplomats in Hispanic America, initially enthusiastic, soon shifted their tone. The diplomatic reports and memoirs of the 1840s and 1850s reflect an increasingly skeptical, anti-republican, and interventionist language. Political chaos, caudillo personalism, the supposed moral decadence of the republics, and the mistreatment of Spanish emigrants were frequent complaints ^79^. Many diplomats, merchants, and emigrants called for a permanent naval presence by the Spanish Royal Navy to guarantee the safety of Spanish subjects and protect the metropolis’s commercial interests. Peaceful pan-Hispanism thus gave way to a securitized and aggressive version that prioritized hard power instruments ^80^.

This ideological evolution had significant political consequences. Beginning in the 1840s, the moderate wing of Spanish liberalism—the same faction advocating for a censitary and centralized order in the Peninsula—began to view external projection as a way to reinforce the authority of the conservative constitutional monarchy ^81^. An interventionist doctrine took shape, calling for the protection of overseas Spaniards, promoting friendly governments in the Spanish-American republics, and using naval power as a diplomatic tool. Spain was to act as a stabilizing force within the Hispanic world, exporting its model of constitutional monarchy and leveraging its limited but growing military capacity to command respect ^82^.

The failure of the pacifist version of pan-Hispanism thus gave way to an emerging strategic doctrine that displaced the Progressive Party subculture’s vision, elevating the interventionist pan-Hispanism promoted by the leaders of the Moderate Party. This strategic subculture would remain hegemonic until 1868. It did not entirely eclipse its progressive and republican competitors but had the most significant influence on state policy ^83^. As a result, the 1840s witnessed the Ministry of State formally or informally supporting several interventionist initiatives: the monarchist conspiracy in Mexico (1846) ^84^, Juan José Flores’s expedition to install a constitutional throne in Ecuador (1846–1847) ^85^, and negotiations to establish an Franco-Spanish protectorate in Montevideo (1846–1850) ^86^. All these initiatives failed. Spain’s military weakness and institutional instability prevented the launch of serious post-imperial interventions until the late 1850s, as will be discussed further below.

A similar process unfolded in post-Soviet Russia. Following the USSR’s disintegration, the Russian state was forced to redefine its international role. The concept of the “near abroad” (blizhnee zarubezhie), formulated by Evgenii Ambartsumov and other ideologues of the new regime, expressed the aspiration to maintain a sphere of influence over the former Soviet republics ^87^. The Commonwealth of Independent States (CIS), founded in 1991, was to be the vehicle for this integration. It was conceived as a flexible confederation aimed at preserving military, economic, and cultural ties and averting the post-Soviet space’s definitive collapse ^88^.

During the early years of the Yeltsin government, this policy was infused with multilateralism and liberalism. Russia joined international financial institutions, cooperated with the West in resolving regional conflicts, and embraced the principles of cooperative diplomacy ^89^. The goal was to ensure a peaceful transition to capitalism and political pluralism while keeping the former Soviet republics within a geostrategic orbit that would not compromise Russian security. In this context, the CIS represented a promise of amicable continuity.

Nevertheless, this promise soon broke down. The CIS failed as a functional structure: its member republics—Moldova, Georgia and Tajikistan—were engulfed in civil wars and began to seek economic and diplomatic autonomy ^90^. Some, such as the Baltic states, decisively reoriented toward the West ^91^. The notion of spontaneous integration based on cultural affinities proved as naïve as the culturalist pan-Hispanism of Spanish liberals. By the mid-1990s, much of the Russian elite had concluded that soft power could not control the near abroad.

This shift was reflected in the rise of Evgenii Primakov, Putin and other advocates of a more assertive foreign policy. Although official rhetoric continued to invoke multilateralism, Russia began to pressure former Soviet republics through energy coercion, support for Russian minorities, intervention in regional conflicts, and an increasingly militarized diplomatic discourse ^92^. Eurasianism—an ideological tendency opposing Western liberalism with a civilizational Russian alternative—gained prominence. Intellectuals such as Aleksandr Dugin developed a geopolitical vision in which Russia was to lead an autonomous bloc grounded in Slavic and Orthodox values, in contrast to the individualistic liberalism of the West ^93^.

The parallel with Spain is striking. In both cases, disillusionment with culturalist projects led to the construction of an aggressive national identity and the rehabilitation of force as a legitimate instrument of foreign policy. The transition from idealistic pan-Hispanism to its interventionist version mirrors the shift from cooperative Post-Soviet internationalism to militarized Euroasianism. The protection of diasporas—Spaniards in the Americas, Russians in Ukraine, Georgia, or the Baltic states—was one of the main arguments used to justify interventionism ^94^. A narrative of external aggression and threats to dispersed national communities allowed for the legitimization of force as a defensive response.

At this stage, Spain and Russia accepted that influence over their former domains could not be rebuilt through cultural persuasion alone. Strategic imposition became the operative logic. The seceded republics were no longer viewed as fully sovereign states but as contested spaces whose political trajectories needed to be shaped by the former metropole. The pretence of a consensual post-imperial community lost importance. Hegemony was to be imposed if it cannot be negotiated.

In sum, Spain’s and Russia’s post-imperial transitions failed in their idealist phases and evolved into doctrines of hard power. The inability to consolidate cultural or diplomatic leadership —combined with internal weaknesses and the hostility of their strategic environments— fueled interventionist projects that laid the groundwork for future wars. As we shall see in the next section, the shift from idealism to bellicism was not instantaneous, but it became inexorable once efforts to restore an empire without imperialism had collapsed.

### 3.3. Paths of reimperialization

Thus, the post-imperial powers under study did not passively accept decline. Neither nineteenth-century Spain nor twenty-first-century Russia regarded the loss of their former dominions as a fait accompli. Instead, both undertook re-imperialization projects —that is, strategies aimed at partially restoring the power they had once exercised during their hegemonic periods. Despite the vastly different contexts, the shared impulse was evident: the need to overcome the geopolitical fragility left in the wake of imperial collapse by restoring prestige, security, and the capacity for international projection. In both cases, this trajectory manifested in a more assertive foreign policy that relied on inherited territorial enclaves and national diasporas as levers to justify and facilitate strategic re-expansion.

In the Spanish case, this policy materialized during the so-called “long government” of the Unión Liberal (1858–1863), led by Leopoldo O’Donnell. After decades of internal instability and diplomatic frustration, Spain found a favorable window for projecting power ^95^. Three factors contributed to this opportunity: relative domestic political stability, an expansive economic cycle fueled by foreign investment and agricultural exports, and an international context shaped by the temporary weakness of the United States, then on the brink of civil war ^96^.

Spanish elites interpreted this scenario as an opportunity to relaunch the pan-Hispanic project through interventionism. This policy’s material basis lay in the Spanish Navy’s modernization. Beginning in 1858 and backed by powerful commercial lobbies based in Mediterranean and Atlantic ports, the Spanish state invested substantial resources in the technological renewal of its fleet. Screw-driven frigates, ironclad vessels, and units capable of sustaining extended overseas campaigns were introduced ^97^. This naval regeneration was not an isolated gesture but rather the central instrument of a broader strategy for international repositioning.

The logic was clear: Spain could no longer aspire to reclaim its former viceroyalties, but it could reemerge as a thalassocratic power capable of dominating maritime routes, protecting its Caribbean and Asian possessions, and exerting pressure on American republics from the sea ^98^. In this new framework, Cuba, Puerto Rico, and the Philippines ceased to be anachronistic vestiges of empire and instead became strategic nodes for the reconfiguration of Hispanic power. In the Caribbean, U.S. expansionism over Cuba and Puerto Rico encouraged a policy of regional containment. In Asia, the Philippines offered privileged access to expanding Pacific trade routes bolstered by the forced opening of Chinese markets. The geopolitical imagination of moderate and unionist Spanish elites increasingly linked the expansion of imperial power with national security ^99^. They viewed overseas presence as vital for the export economy and the Spanish state’s very independence vis-à-vis French and British influence.

This outlook translated into several imperial ventures with initially promising results. The joint intervention with France in Cochinchina (1858–1862) secured privileged access to local labor markets, seen as key to gradually replacing Cuban slave labor ^100^. The invasion of Morocco (1859–1860) did not yield the territorial conquests many had hoped for, mainly due to Royal Navy intervention, which curtailed O’Donnell’s ambitions. However, a treaty was signed that bolstered Spain’s position in the region by granting financial and commercial privileges ^101^. The reannexation of Santo Domingo, undertaken at the request of its government in 1861, would ultimately prove disastrous, but by 1863, it still appeared to have strengthened Spain’s Caribbean foothold ^102^. Finally, Spain’s participation alongside France and Britain in the tripartite intervention in Mexico in 1862 produced unexpectedly favorable outcomes. The ousting of Juárez’s government promised repayment of outstanding debts. Moreover, standing alongside the great European powers gave Spain a sense of restored geopolitical stature. General Prim’s decision to withdraw Spanish forces when it became clear that Napoleon III intended to install a constitutional monarchy was well-received by many Latin American governments ^103^.

The Unión Liberal government also looked to South America with renewed interest. Spanish émigré communities in Peru, Chile, and Ecuador became active agents in re-imperialization. Settled in major cities and involved in local commerce, they began to pressure Madrid for protection amid the political upheavals in their host countries. Complaints included fiscal abuses, forced conscription, and legal discrimination. Some even called for direct Spanish intervention to secure their rights and property ^104^. This victimist narrative was enthusiastically adopted by Spanish diplomats, who transformed it into a moral rationale for interventionism. Power projection was to be framed not as conquest but as protection ^105^. Gunboat diplomacy was presented as a civilizing response to republican disorder. At the same time, intervention would enable favorable treaty-making, the defense of commercial enclaves, and potentially promoting conservative, pro-Spanish governments ^106^.

This opinion campaign culminated in the creation of the Pacific Squadron in 1862. Its goal was nothing less than to signal Spain’s reemergence as a hemispheric power. The carefully planned expedition was to visit major South American ports on both the Atlantic and Pacific coasts, protect Spanish nationals, promote trade agreements, and, if possible, establish a permanent naval base in Ecuador ^107^. This initiative would ultimately lead to the Spanish-South American War, as discussed in the following section.

Though distinct in temporal and geographical coordinates, the Russian case largely mirrors this strategic pattern. Following the failures of the Commonwealth of Independent States (CIS) and the frustrations of cooperative Eurasianism, Putinism embraced a logic of imperial re-expansion based on three elements: control over the "near abroad," protection of Russian minorities in neighboring countries, and the strengthening of military power as a key tool of foreign policy ^108^. The shift from post-Soviet idealism to post-imperial interventionism reached a turning point in the mid-2000s when it became evident that the West would not respect the sphere of influence that Moscow considered vital.

The 2004 NATO enlargement, incorporating several former socialist bloc countries, including the Baltic states, was perceived as a direct threat. The subsequent expansion of the European Union in the same direction reinforced this sense of encirclement. Simultaneously, pro-Western social movements in Georgia, Ukraine, and Kyrgyzstan—the so-called "color revolutions"—threatened to install governments hostile to Moscow in key post-Soviet states. The prevailing perception in the Kremlin was that Russia risked geopolitical encirclement, marginalization, and fragmentation if it did not act swiftly ^109^.

Thus, Putinism initiated a grand strategy to securitize its immediate environment. This strategy encompassed the containment of Western advancement and protecting Russian or Russian-speaking communities residing in former Soviet republics. The argument paralleled Spain's: these minorities were being harassed, discriminated against, or deprived of their rights, and only the intervention of the mother state could ensure their well-being ^110^. Foreign policy adopted a hybrid form, combining coercive diplomacy, intelligence operations, covert military presence, and direct actions ^111^.

Georgia became the first theater of this forceful reimperialization. In 2008, capitalizing on a latent conflict in the separatist regions of South Ossetia and Abkhazia, Russia intervened militarily and consolidated its control over both territories. From then on, these functioned as de facto Russian protectorates. It was a surgical operation, limited in time but effective in its objectives. Not only did it neutralize a hostile government in Tbilisi, but it also demonstrated to the entire near abroad that Russia was willing to use force to defend its sphere of influence ^112^. The analogy with Spain's naval projection in South America is revealing: both states acted in weakened environments, exploited local disorder, and legitimized their actions with the language of protection.

The next major theater for interventionist Eurasianism was Ukraine. After the USSR's collapse, Ukraine and Russia maintained relatively stable relations, marked by pro-Russian Ukrainian governments during the 1990s. However, the situation changed in 2004 with the Orange Revolution, which brought the pro-Western Viktor Yushchenko to power. From then on, Ukraine became politically divided between "orange" sectors, favorable to NATO and opposed to Russian influence, and "blue" sectors, advocating for a Ukraine closer to Moscow and defending the rights of the Russian-speaking population ^113^.

The rise of Viktor Yanukovych in 2012 temporarily restored ties with Russia, but the Maidan protests in 2013–2014, driven by social discontent and pro-Western sectors, overthrew his government. This instability prompted Russian intervention: in February 2014, pro-Russian militias took control of Crimea, which Moscow subsequently annexed. Simultaneously, conflicts erupted in Luhansk and Donetsk, where pro-Russian militias began demanding autonomy and language guarantees, covertly supported by Russia. The election of Petro Poroshenko as president in May 2014 hardened Kyiv's stance, refusing to negotiate with the rebel territories. Although these regions were not militarily reconquered, a frozen conflict ensued. The arrival of Volodymyr Zelensky in 2019 intensified the Ukrainian government's anti-Russian tone and accelerated its rapprochement with NATO ^114^.

All this occurred, as mentioned, in a context where Russia, under Putin's leadership, was asserting itself as an interventionist post-imperial power. Successful campaigns in Georgia and Crimea, the subordination of Belarus to client-state status—through mechanisms of soft power and informal imperialism—and power projection in theaters like Syria ^115^ and the Sahel—whether through official aid or via the Wagner Group—reinforced the strategic culture of Putinist Eurasianism without Russia facing significant reprisals from the West.

The economy played a central role in the Spanish and Russian cases. The oil and gas boom from 2005 onwards enabled Russia to finance its rearmament, modernize parts of its armed forces, and gain maneuvering room on the international stage. Like Spain in the 1850s, Russia experienced a boom cycle coinciding with an internal consolidation of executive power. Putinism articulated a stable authoritarian order backed by high popularity ratings and a divided and weakened opposition. This combination of economic resources and political cohesion fueled reimperialization ^116^.

Another significant parallel lies in how the perception of weakness in the strategic adversary reinforced these policies. Spain interpreted the American Civil War as a sign of Anglo-Saxon decline ^117^. Russia, for its part, viewed the leadership crisis in the West following the Iraq war, the rise of Donald Trump, and the chaotic withdrawal from Afghanistan as symptoms of irreversible decay. In both cases, the post-imperial powers became convinced that it was time to act, to make up for lost time, and to reposition themselves before geopolitical conditions changed again.

A final common element in both re-imperialization processes is worth mentioning. Both the Unión Liberal (1858–1863) and Putinism in its second stage (2008–2025) bet on imperial nationalism as a symbolic instrument of internal cohesion. The re-expansion campaigns in Asia, the Caribbean, Morocco, and the Pacific articulated a discourse celebrating Spain's geopolitical regeneration ^118^. This garnered support from a broad sector of liberalism, as well as from military establishments and Spanish mercantile capitalism, creating a consensus climate that promised to end the internal divisions following the imperial collapse, turning Spain into a consolidated state ^119^. Nationalism, militarism, and post-imperial imperialism reinforced each other, institutionalizing a language of legitimacy that strengthened the strategic culture of interventionist pan-Hispanism. Something similar has occurred in Putinist Russia, where anti-Westernism and irredentism have contributed to weaving a narrative of imperial restoration that has achieved notable success in public opinion ^120^. Post-imperial Eurasianism has served as a substitute for civic nationalism, which is challenging to sustain given the regime's authoritarian dispositions, and for ethnic nationalism that struggles to reflect the ethnic diversity of Russia and the post-Soviet space ^121^.

Thus, both Spain and Russia embarked on reimperialization, convinced that post-imperial interventionism could be the fulcrum to regain international relevance and consolidate their internal political orders. In both cases, re-expansion was articulated around three elements: territorial security, protection of diasporas, and exploitation of an international context perceived as favorable. As we will see in the following section, this restoration strategy would clash with the military and political reality of the chosen scenarios, leading to wars whose outcomes would test the true capabilities of the powers in question.

Between October 1862 and April 1864, the Pacific Squadron sailed along the coasts of Brazil, the Southern Cone, Chile, Peru, Ecuador, Central America, and California, with the mission of projecting the influence of the Spanish Monarchy through a naval show of force. This first circumnavigation yielded initial diplomatic successes and appeared to rekindle the idea of a regenerated Spain in the eyes of Spanish America ^122^. However, in 1864, citing grievances against Spanish citizens in Peru, Commander Pinzón ordered the occupation of the Chincha Islands—then, as noted earlier, a strategic reserve of guano. Far from a miscalculation, this action aligned with the Union Liberal’s plan to impose an unequal treaty on Peru utilizing gunboat diplomacy.

The Vivanco–Pareja Treaty, signed in 1865, enshrined Spain’s objectives: compensation for naval expenditures, recognition of viceregal debts, restitution of confiscated properties, and the granting of commercial and judicial privileges. It appeared that a limited intervention had achieved its goals. However, the public reaction in neighboring Chile was swift and forceful. An anti-Spanish campaign driven by radical factions pressured the Santiago government—until then neutral—to obstruct the provisioning of the Spanish fleet. This event precipitated a declaration of war by Spain against Chile and, following the collapse of the Peruvian government, against Peru as well ^123^.

By 1865, Spain already suffered from political instability, economic crisis, and internal divisions. The growth cycle initiated in 1856 had proven highly volatile, resting more on a financial bubble than on any structural modernization of the economy ^124^. In this context, the Squadron—lacking secure ports or logistical support—sought a confrontation with the Chilean-Peruvian fleet to preserve its deterrent image. The aim was to safeguard national prestige through a punitive strike on enemy ports. The inconclusive battles at Papudo and Abtao, coupled with the loss of the Covadonga, led to the suicide of Admiral Pareja—who had succeeded Pinzón—and the rise of Méndez Núñez, who opted for the bombardments of Valparaíso and Callao as a last resort ^125^.

International public opinion coldly disapproved of the bombardment of Valparaíso on 31 March 1866: Chile offered no resistance, and the attack on a defenseless port was roundly condemned. The assault on Callao on 2 May was more destructive but depleted the Spanish fleet. Upon its return, the Spanish press and government attempted to present the attacks as a victory, portraying them as evidence of Spain’s resurgence as a hemispheric power. Nevertheless, this narrative failed to gain traction abroad. Foreign powers condemned the damage to commercial interests in Valparaíso and Callao as acts of barbarism ^126^. The reaction in South America was fierce: attacks on Spanish immigrants and widespread expropriations followed, bankrupting many. The Monarchy, unable to dispatch reinforcements, was in a state of terminal decline. The Glorious Revolution of 1868 ended continuity in Spain’s foreign policy and ushered in a new transformation of its strategic culture, which we will examine in the next section. The 1871 armistice sealed a symbolic defeat: post-imperial interventionism had undermined Spain’s prestige and influence on the continent ^127^.

This episode presents striking parallels with Russia’s intervention in Ukraine beginning in February 2022. In both cases, a post-imperial power believed it could exert influence over a former territory at little cost. Russia’s so-called “special military operation,” like the occupation of the Chinchas, was not preceded by a formal declaration of war. Both actions were presented as humanitarian measures to protect nationals or cultural kin from alleged violations of the civilized order ^128^.

The language used obscured broader strategic aims: Spain sought to contain U.S. influence in the Pacific, secure access to guano, and establish a naval base. Russia aimed to halt NATO expansion, control energy corridors, and reassert its hegemony in the “near abroad.” Both projects were founded on the assumption that a limited show of force would suffice to impose their terms. Spain nearly succeeded with the Vivanco–Pareja Treaty. In its most optimistic scenario, Russia expected to replace Ukraine’s government with a friendly regime through a limited military deployment. However, both wars exceeded their planners’ expectations. In Spain’s case, the protracted conflict exposed its logistical weaknesses, the lack of international support, and the vulnerability of its expatriates. In Russia, although significant territorial gains have been made, the war has devolved into a costly war of attrition, exacting a high human, economic, and political toll.

Although it is too early to predict the war's outcome in Ukraine, the Spanish case offers a cautionary tale about the pitfalls of post-imperial interventionism. At first glance, both powers appear to have underestimated their adversaries, overestimated their ability to impose order, and lost control of the international narrative.

## 4. Results

### 4.1. Postimperial patterns: collapse, failure, soft reimperialization, hard reimperialization

The disintegration of the Spanish and Soviet empires was followed by the partial breakdown of their respective core territories. Both Spain and Russia were political formations with high degrees of “imperiality”; their institutional and power structures were deeply dependent on the integrity of their imperial systems. As Lachmann, following Maier, would argue, Spain and Russia did not merely “possess” empires—they “were” empires ^129^. Their political institutions, fiscal and administrative apparatuses, and economic models were fully amalgamated with the imperial systems of which they were part. The collapse of the Catholic Monarchy after the Napoleonic Wars and that of the Soviet Union following its implosion in 1991 were not simply processes of decolonization affecting external dependencies. Instead, they entailed the rupture of the ideological and institutional foundations that had given coherence to their core territories. The corporative system of the Ancien Régime and Soviet communism were mortally wounded. Thus, neither the Spanish Monarchy nor the Russian Federation emerged as fully consolidated nation-states. Instead, they remained “fragments of empire” mired in dysfunction ^130^. The decades following their imperial collapses were marked by internal decomposition and political anomie both in Isabelline Spain and post-Soviet Russia.

These imperial dissolutions were also sociopolitical revolutions that affected all parts of the former systems. Both the Hispanic and post-Soviet spaces experienced the deinstitutionalization and disorganization of the networks of power that had prevailed during the long imperial periods that had shaped them ^131^. It follows that the degrees of rupture from the imperial past were particularly radical in the post-revolutionary moment. Spanish and Spanish American constitutionalists entertained a wide range of transformative and even utopian political horizons aimed at de-imperializing their societies by dismantling the preexisting institutional frameworks through representative government and economic liberalization ^132^. In the post-Soviet world, hopes arose that democracy and capitalism would exert a modernizing influence ^133^.

However, in practice, the immediate ruptures of the empires did not correspond to these ideological possibilities, particularly in the cases of Spain and Russia. The critical junctures of the 1830s and 1990s proved catastrophic: both regions experienced prolongations of imperial collapse through the Carlist and Chechen wars, accompanied by institutional chaos and political polarization. These experiences of internal decline triggered a new historical sequence: they led to the institutionalization of strategic cultures premised on the idea that creating spheres of influence over former dependencies was key to geopolitical revival, internal stability, security, and sovereign survival. Within these strategic cultures, grand strategies of post-imperial regeneration emerged, crystallizing ideologically in the doctrines of pan-Hispanism and Eurasianism, which guided state action. In other words, imperial ruptures did not give rise to stable post-imperial sovereignties. The traumatic and unresolved nature of the conflicts stemming from the imperial collapse gave rise to strategic cultures that returned to “imperiality” or “imperial tradition” ^134^ as a regenerative horizon, reactivating the geopolitical agendas inherited from the eighteenth-century Bourbon imperial culture and its Russian counterpart, rooted in Tsarism.

Other relevant factors must be added to all this. Political and military elites were not the only architects of the new strategic frameworks. Diasporas of Spaniards and Russians scattered across former imperial dependencies often encouraged the idea of fostering post-imperial spheres of influence, sometimes motivated by a desire to recover the status quo they had lost with independence. Likewise, multiple economic power networks were interested in imperial reconnection. For instance, commercial consulates from the Peninsula—particularly those in Cádiz and Barcelona—had depended on the imperial monopoly system for a significant portion of their profits. These business actors consistently promoted the pan-Hispanic agenda. In the post-Soviet space, energy markets and the dependencies created under the USSR became key strategic instruments for extending Russian influence in the Near Abroad ^135^.

In short, high degrees of “imperiality” in both cases led to violent and anarchic collapses. These threatened numerous entrenched interests and stimulated the recycling of old imperial strategic cultures. The critical junctures described above reinforced, in new forms, the path-dependent trajectories inherited from the disintegrated empires. The uncertainty generated by the failure to consolidate non-imperial states, markets, and ideological frameworks during the 1830s and 1990s gave rise to nostalgic imperial memories, which intertwined with forward-looking expectations oriented toward re-imperialization ^136^.

In their initial phase, both post-imperial grand strategies relied on soft power, seeking to institutionalize post-imperial communities through diplomacy and cultural influence. The cooperative and pacifist form of pan-Hispanism aligned with the strategic subculture of the Spanish Progressive Party, while Eurasianism of a similar nature emerged in a context in which Moscow’s elites remained open to collaboration with the geopolitical West. However, the constraints of the international system and the limited capabilities of Spain and Russia led to the rise of emergent strategies that altered the course of their grand designs. A new critical juncture emerged in the 1840s and the 2000s, producing new sequences characterized by the interventionist and militaristic turn of imperial strategic cultures—now promoted by the Spanish Moderate Party and Putinism. In both cases, a strategic subculture prioritizing hard power—through military projection and interventionism—gained ascendancy as the preferred method for constructing post-imperial spheres of influence. Fueled by miscalculations, these developments triggered wars with the successor states central to pan-Hispanic and Eurasian schemes.

The post-imperial pattern described for both cases thus follows the following sequence: (1) imperial collapse; (2) failure to construct a prosperous post-imperial state and economy; (3) elaboration of a post-imperial regeneration strategy based on soft power; (4) failure and limitation of said strategy; (5) re-elaboration of the regeneration strategy along militaristic and interventionist lines; (6) aggressive re-imperialization policies and popularization of imperial nationalisms.

### 4.2. Th historical structures of reimperialization

In order to assess in greater depth the causal chains that conditioned these patterns, it is necessary to turn to theories developed in the fields of political science and comparative historical sociology of empires. In this regard, the tripartite framework proposed by Doyle for the dynamics of imperial construction seems particularly useful. According to him, a structuralist explanation of imperial expansion must combine three interpretations: the metrocentric, the pericentric, and the systemic. That is, it must explain which factors account for the willingness of a political community to expand beyond its borders (metrocentric); which factors account for why a political community is susceptible to being controlled by another, whether through informal or formal mechanisms (pericentric); and which factors account for whether the international arena is favorable or unfavorable to imperialist actions (systemic) ^137^.

We believe this model applies to re-imperialization processes and complements well the model outlined in the previous section, since the levels of imperiality, degrees of rupture, and processes of institutionalization of imperial strategic cultures would influence both the configuration of the former metropole and its ex-dependencies, as well as their positioning within the international system. We also draw on the work of a little-cited historian and political scientist, Alexander John Motyl. In his book Imperial Ends: The Decline, Collapse, and Revival of Empire —which predicted Russia’s aggressive re-expansion as early as 2001—he dared to adapt Doyle’s theories to the dynamics of re-imperialization, combining them with other recent works in the comparative sociology of empires ^138^.

One of the most significant variables identified by Motyl in his theorization of imperial revivals concerns temporality. The author emphasizes the importance of the pace of imperial disintegration. In his view, imperial formations that do not disintegrate gradually but suffer sudden collapse are more likely to leave behind conflictive post-imperial scenarios. Once the war, political crisis, or natural disaster that caused the sudden disintegration has passed, the former imperial core may recover elements of internal power that enable it to re-establish its former authority, wholly or partially ^139^. Furthermore, we might add —following Kaplan— ^140^ that abrupt, unplanned ends prevent the institutionalization of a framework of post-imperial relations prior to the rupture. This hinders the ability of strategic cultures within former imperial cores and elite power groups to formulate non-imperial doctrines regarding security, their international role, and their position in the global market.

The turbulent end of the Spanish viceregal system in the Americas and that of the Soviet Union correspond to this typology of collapse. Neither of these empires experienced an organic process of territorial disintegration. Although long-term internal tensions marked both, they showed no immediate signs of dismantlement until they “burst apart” due to disruptive phenomena: the Napoleonic Wars in the Spanish case, and the collapse of communism in the Soviet case. Motyl argued that, in such situations, if the imperial core managed to achieve a sufficient degree of internal reconstitution, it tended to attempt to reassert its authority over its former domains ^141^ —as was the case, for example, with Nazi Germany or the seventh-century Byzantine Empire.

The comparative narrative developed in the previous sections supports Motyl’s theory. As soon as Spain and Russia partially recovered from the cycles of internal disorganization that accompanied their respective geopolitical collapses, they implemented post-imperial regeneration strategies that led to the wars under study. Neither the seizure of the Chincha Islands nor the invasion of Ukraine were ministerial improvisations; rather, both were expressions of structural patterns of re-imperialization. In both cases, powerful groups within the state and sectional elites had strong incentives to dream of re-establishing imperial power networks, particularly after having experienced their collapse as a cycle of disorder and impoverishment.

However, beyond the structural symmetry we may infer in their causes, the Hispano–South American War and the Russo–Ukrainian War differ significantly when analyzed through Motyl’s framework. The American political scientist argued that post-imperial irredentisms are successful when several conditions coincide. The most favorable scenario for re-expansion occurs when a well-institutionalized, stabilized, and powerful former imperial core seeks to reconquer or indirectly control a former dependency that has failed to stabilize its post-imperial sovereignty, is mired in legal anomie and internal division, and lies outside the sphere of influence of another great power ^142^.

We would venture that the case of Spain and the Hispanic American republics diverged more significantly from this model than Russia and Ukraine. It is true that, after the end of the First Carlist War, the Spanish monarchy experienced a period of relative stability under the political hegemony first of the Moderate Party (1844–1854) and then of the Liberal Union (1858–1863). However, we should be under no illusions. The censitary and centralist constitutional system enshrined in the Constitution of 1845 led to the formation of clientelist factions that exercised power exclusively, marginalizing the political opposition and failing to garner sufficient social support. This left Madrid’s governments perpetually threatened by the possibility of military pronunciamientos or popular uprisings. The appearance of normalization brought about by the “Long Government” (1858–1863) led by Leopoldo O’Donnell proved to be a mirage. Chronic divisions re-emerged after his fall, culminating in the collapse of the constitutional regime of 1845 and the Isabelline monarchy in the revolution of 1868, ending the state’s institutional continuity.

These abrupt political cycles had their economic counterpart. O’Donnell’s government coincided with a cycle of euphoria driven by the boom in agricultural exports and Anglo-French investments. However, the foundations of that early phase of Spanish capitalism soon proved fragile and insufficient. When the investment cycle slowed and global trade decelerated in the 1860s, an economic crisis ensued that prevented Spanish governments from allocating budgetary resources to renew the Spanish Royal Navy and to the projection of overseas power. This weakness of the old imperial core explains why Spain could not sustain its war effort beyond the punitive acts represented by the bombardments of Valparaíso and Callao. This rendered its aggressions mere expressions of impotence, rather than credible demonstrations of a gunboat diplomacy capable of being sustained over time. The imperial nationalism that had served as a cohesive force during the Liberal Union’s period of hegemony collapsed along with the institutional disintegration of the state and the failures abroad.

Moreover, Spanish diplomats and ministers misjudged the strength of the Pacific South American republics. It is true that the initial target of the intervention, Peru, did not appear particularly powerful in 1864. Its economic growth since 1848 was noteworthy but almost entirely based on guano exports. This made the Republic particularly vulnerable to a naval attack that could deprive it of its reserves of this fertilizer, the most important of which were located in archipelagos such as the Chinchas. The Peruvian navy was far inferior in number and combat capability to the Spanish warships deployed to its coast. Additionally, the civil conflicts and uprisings that had pitted liberal and conservative factions against one another over the preceding decade led Spanish diplomats and naval officers to expect a weak government in Lima, especially if deprived of the revenues derived from guano export duties.

These calculations were not entirely mistaken. In fact, the signing of the Vivanco–Pareja Treaty in January 1865 seemed to confirm them. The error in Spain’s grand strategy lay with another country: Chile. The Spanish monarchy’s ministerial planners had assumed that the southern Republic would not intervene in any international dispute detrimental to Peru. After all, Valparaíso and Callao competed for hegemony as the dominant entrepôt of the South Pacific. The War of the Confederation (1836–1839) had already revealed the geostrategic rivalry between the two countries, consolidating the image of Spanish America as a disunited region in which every attempt at alliance or confederation was thwarted by the particularistic interests of its republican leaders. Based on these premises, no one in Madrid expected Santiago to support Lima.

In this regard, the Spanish elites made two fatal interpretive mistakes. First, they failed to anticipate that the recent wave of European interventionism in the Americas had galvanized democratic radicalism across the continent, committing a sector of Chile’s intellectual and political elite to the cause of Americanism. Second, they did not foresee that Chile’s ruling class conceived of their state as a regional power that sought to maintain a balance of power favorable to its position as the commercial hub of the Andean coast. Chile’s entry into the conflict contributed to a change of government in Peru, the outbreak of war, and the formation of an alliance with Ecuador and Bolivia that cut off the Spanish fleet’s supply lines and any possibility of securing a port base. Although the Royal Spanish Navy had clear military superiority, the Chileans and Peruvians prevailed on logistical and diplomatic fronts, thereby thwarting Spain’s attempt at geopolitical regeneration.

A former imperial core weaker than it appeared was defeated by two former imperial dependencies that proved more vigorous and strategically ambitious than initially expected. In this respect, it is crucial to highlight the role of the strategic culture of Americanism, forged during the wars of independence, which advocated a defensive alliance among republican America against any European interventionist attempt. The failure of Spain’s grand strategy led to twenty years of stagnation in trade and migration links with both countries and contributed to the consolidation of Chile’s regional hegemony. Just fifteen years later, Chile would annex substantial territories from Bolivia and Peru and establish naval dominance over the region. Only the return to a post-imperial strategic culture based on soft power and cultural diplomacy allowed Spain to reconnect with its former imperial domains successfully.

The form of Hispano-Americanism that emerged between 1876 and 1936, despite the weaknesses of the Spanish state, promoted academic cooperation, the ritual celebration of Hispanic identity in the face of U.S. expansionism, and the search for economic complementarities. This was accompanied by phenomena such as the mass migration of Spaniards to Latin America, the renewed presence of Spanish clergy, and the creation of dense transatlantic circuits of intellectual and cultural exchange ^143^. These developments had long-term effects, explaining the important benefits that certain power groups and sectors of Spanish society in the 21st century still derive from post-imperial relations ^144^.

In this respect, the Russian case is markedly different. Since Putin came to power, political stabilization and economic consolidation have proven far more robust than those experienced in Isabelline Spain. Putinism has pulled the Russian Federation out of the total disintegration and decline that plagued it during the 1990s. The authoritarian democracy that has taken shape has managed to subordinate the projects of the economic oligarchy, at least partially, to the imperatives of the state, while securing the support of broad sectors of the middle and lower classes, whose quality of life has improved over the past two decades. Although Russia’s economy remains highly dependent on energy exports, the country has also become competitive in agricultural production. It has retained a degree of autonomy in technological sectors such as arms manufacturing and computing.

While Putin’s Russia has entered the circuits of global trade and finance, it has done so through calculated protectionism and a diversification of trade partners that shields it from the geopolitical hegemony of the West. This explains the limited impact of the sanctions imposed by the EU and the United States after 2022. The controlled authoritarian democracy established by Putin has secured high levels of popular support, which, combined with the ideological promotion of anti-Western Eurasianism and the effective repression of dissent, has granted him a degree of control over the political system far more significant than that enjoyed by the Liberal Union of Leopoldo O’Donnell.

The great weakness of post-Soviet Russia remains demographic. The continuous decline in birth rates is leading to an inevitable reduction of the Russian population. However, we must note that this factor has not discouraged post-imperial interventionism under Putinism; instead, it has served as an incentive, as Moscow prefers to act militarily before its human resources are further diminished by population aging. This leads us to conclude that the risk of conflict increases when a post-imperial power links the resolution of its internal crises to imperial regeneration rather than to other possible reformist or redistributive agendas. In such cases, post-imperial weakness in certain domains may actually become a driver of re-imperialization processes.

Ukraine, for its part, has proven to be a more formidable adversary than Russian planners initially expected. Unlike Peru, it has not benefited from the intervention of unexpected allies on its borders, but it has received considerable military support from NATO. In any case, Ukraine’s erratic post-Soviet history made it appear unlikely that the country would show either the military or political resilience to withstand the initial Russian assault. After all, no Ukrainian government—not even that of Volodymyr Zelensky—had established a level of control over the oligarchies comparable to Putin’s. The deep partisan and ethno-linguistic divides that, since the 1990s, have separated the Russophone east from the center-west of the country have only worsened since the loss of Crimea and the implementation of anti-Russian assimilation policies.

Yet precisely this latter vulnerability ultimately contributed to Ukraine’s wartime resistance after the 2022 invasion. The cohesion of the country’s economic-political elites and of the populations in the central and western regions has been reinforced by intense anti-Russian nationalism and the war itself. This has enabled the organization of an effective resistance that has prolonged the conflict.

However, Ukraine faces certain clear disadvantages when compared to the situation of Chile and Peru during the Hispano–South American War. To begin with, Ukrainians have their nemesis at their doorstep. The conflict is taking place along the extensive land border the country shares with Russia, and it is precisely in the border regions that the Russophone diaspora, who supported the intervention, is concentrated. This allows Moscow to deploy ground forces quickly, carry out continuous bombardments of Ukrainian territory, and maintain logistical supply lines. It is also no small matter that Belarus, as a satellite state of Putinism, permits the invading army to operate from within its sovereign territory.

Here, we find a key point of divergence between the two post-imperial conflicts. Isabeline Spain could not deploy troops in the Southern Cone. It lacked the naval and logistical means to carry out an amphibious operation in a region so distant from Cuba and the Philippines. Nor did it have allies capable of providing a territorial base and supply point. That had been the initial plan with Ecuador, whose government had given certain guarantees to the Liberal Union that it would support the establishment of a Spanish naval base in Guayaquil. However, Chile’s entry into the war changed the initial balance of power and prompted the Ecuadorian government of Gabriel García Moreno to align itself with the Americanist cause. Deprived of supply points along the western coast of South America, the Spanish Pacific fleet was left stranded. Hence its desperate pursuit of a quick confrontation and eventual resort to port bombardments.

The Soviet Union was a compact, land-based empire; the Spanish monarchy, by contrast, was an overseas empire separated from its colonies by vast oceanic distances. These geographical determinants played a decisive role in the post-imperial irredentisms under discussion. Russia can project power over its “near abroad” far more forcefully than Spain could over the immense and distant countries that broke away from its Crown. Suppose we engage in counterfactual thinking and imagine a scenario of geographical contiguity between the Iberian Peninsula and South America. In that case, it is easy to suppose that the government of the Liberal Union would have deployed its not-insignificant land forces in Chile and Peru, let alone possessed the means to sustain its naval aggression over time. Such a situation would likely have altered the outcome.

Beyond geographical imperatives, the international context in which both conflicts unfolded was markedly different. It is true that the rival powers—namely the United States and the British Empire—displayed notable passivity during the Hispano–South American War. The Chilean government under Álvaro Covarrubias expected that, when Méndez Núñez’s squadron prepared to bombard Valparaíso in March 1866, the U.S. and British fleets stationed in the port would defend it in the name of commercial interests and international legality. However, the United States was then in the throes of its post–Civil War reconstruction, while Britain was wary of becoming entangled in a conflict that could create friction with its European allies. The South American Pacific republics found themselves alone in the face of Spain, but they succeeded in forging a solid alliance among themselves. The strategic culture of Americanism, born out of the continental and imperial rather than national character of the independence wars, served as a catalyst for the adhesion of Chile, Ecuador, and Bolivia to a cause that, initially, concerned only Peru. One could point to more particularistic interests behind the decisions of their respective governments, each of which acted in line with its continental power aspirations. Be that as it may, the Spanish Pacific squadron faced a solid bloc of states that blocked its access to the continent and its ports.

The post-Soviet states, by contrast, lack a strategic culture conducive to cooperative resistance against Russian power comparable to Americanism. Their regimes are not the product of a war of independence, and, except for the Baltic republics, they have not built solid institutional structures for multilateral cooperation. Moreover, over the past two decades, Putin’s Russia has skillfully employed more subtle instruments of power, such as energy and technological cooperation or support for autocratic leaders, to create a sphere of influence among those states less resistant to its hegemony than Ukraine. As Alexander Cooley has explained, the resort to militarism and intervention in places like Georgia or Ukraine has not prevented Putinism from simultaneously pursuing softer re-imperialization strategies in the rest of the post-Soviet space. In this respect, it has been remarkably successful—unlike Isabeline Spain, which failed to institutionalize regular mechanisms for projecting soft power in the years leading up to the Peruvian intervention, allowing the Americanist reaction to go unchecked.

Thus, despite appearances, Ukraine has encountered a more hostile international theatre for its defense than Peru or Chile once did. While the geopolitical West has offered it declarative support and essential military aid, the United States has—at least until the re-election of Donald Trump—adopted a more assertive stance than it did during the Hispano–South American War, though without entering into a formal alliance or mobilizing NATO in full. Even the provision of military and economic assistance remains subject to the volatility of U.S. domestic politics and the perennial disagreements and strategic limitations within the European Union. Unlike the Peruvian or Chilean governments at the time, the Zelensky administration lacks committed allies along its borders. As we have already noted, the absence of wars of independence in the post-Soviet space resulted in very low degrees of rupture, preventing the emergence of a strategic culture comparable to Americanism.

On the other hand, the confrontation between Spain and the South American republics took place during a period of U.S. lethargy, but that Civil War–induced pause was only temporary. Afterwards, the United States resumed its unstoppable rise toward continental hegemony, culminating in the Spanish–American War of 1898. At the same time, the strength of the Portalian regime in Chile ensured that this once-peripheral former colony consolidated itself during the 1870s and 1880s as the most potent maritime and military power in the South Pacific.

In short, Spain’s strategic objectives in its intervention in Peru ran counter to the geostrategic trends of the region and the emerging global power structure that would come to fruition in the second half of the nineteenth century. In the Russian–Ukrainian case, we lack the benefit of hindsight. It is difficult to predict what will happen, but all signs suggest that U.S. global hegemony is not merely in a period of dormancy but rather in active decline. The multipolar order now taking shape—driven by China’s rise and the assertiveness of regional powers such as Turkey, India, and Iran—has allowed Russia’s aggression to be met with acquiescence from a significant portion of the world beyond NATO. Unlike the Hispano–South American War, the post-imperial conflict between Russia and Ukraine may well be symptomatic of a broader reconfiguration of global geopolitics—this time involving the erosion of U.S. and Anglo-Saxon influence.

### Conclusion

This article has proposed a comparative analysis of the reimperialization processes of Liberal Spain (1833–1868) and post-Soviet Russia (1991–2025), focusing on how both powers, following the sudden collapse of their respective empires, developed strategic cultures inclined toward recovering influence over their former imperial domains. Drawing on historical sociology, strategic studies, and institutionalist theories, we have argued that the failure to consolidate internally stable, ideologically coherent, and geopolitically secure post-imperial states led both Spain and Russia to formulate grand strategies of regeneration centered on the restoration of imperial power, whether symbolic or material.

Our theoretical framework introduced the notions of “levels of imperiality” and “degrees of rupture” to assess the extent to which post-imperial societies remained structured by inherited imperial institutions and logics. In both Spain and Russia, the disintegration of their empires was not accompanied by a definitive political re-foundation. Instead, the collapse generated chaotic transitions marked by civil wars (Carlist and Chechen), political anomie, and the recycling of imperial imaginaries as tools for internal stabilization. This gave rise to post-imperial strategic cultures that sought to create spheres of influence in their former peripheries, initially through soft power and later through increasingly coercive instruments.

In both cases, the soft phase of post-imperial diplomacy—pan-Hispanism and post-Soviet internationalism—was short-lived. The limited effectiveness of diplomatic and cultural outreach, coupled with internal weaknesses and international frustrations, prompted a shift toward hard power strategies. In Spain, the Moderate Party’s embrace of interventionist pan-Hispanism culminated in the ill-fated Hispano–South American War. In Russia, the evolution of Eurasianist thought under Putinism laid the groundwork for a sequence of interventions in Georgia, Crimea, and ultimately the full-scale war in Ukraine. In both scenarios, the protection of diasporas and the restoration of national prestige served as ideological justifications for military action.

The comparison also highlights key differences in outcome and global resonance. Spain, despite its ambitions, lacked the logistical capacity, internal cohesion, and geopolitical leverage to sustain its interventionist projects. The Spanish defeat in the Pacific revealed the limits of its reimperialization and marked the beginning of a return to soft post-imperial diplomacy. Russia, by contrast, has demonstrated greater resilience in projecting force and consolidating a sphere of influence, even in the face of international sanctions and military setbacks. Its reimperial trajectory has benefited from geographic contiguity, demographic weight, and a favorable multipolar global environment that limits Western capacity for unified response.

From a broader perspective, this article suggests that post-imperial conflicts are best understood as historically patterned sequences rather than isolated contingencies. The proposed six-stage model—from imperial collapse to militarized reimperialization—captures the structural dynamics that recur across different post-imperial contexts. Furthermore, by integrating metrocentric, pericentric, and systemic variables, we have shown that the success or failure of reimperialization efforts depends not only on the strategic will of the former imperial center, but also on the internal configurations of the successor states and the permissiveness or resistance of the international system.

Ultimately, the comparative study of Liberal Spain and post-Soviet Russia challenges linear and teleological views of decolonization. It reveals that empire does not simply end with political independence or territorial loss. Instead, imperial legacies persist in the institutional frameworks, strategic imaginations, and political calculations of both former metropoles and successor states. Recognizing these patterns is essential for understanding the resurgence of imperial logics in the twenty-first century—and the risks they pose for global stability.

## Ethics and consent

Ethical approval and consent were not required.

## Data Availability

This article does not include original datasets, as it is conceived as a theoretical, methodological and bibliographic essay. Its aim is to develop conceptual tools and interpretive frameworks through a comparative reading of historical cases, drawing primarily on existing scholarship. All sources cited are publicly available, and no new empirical data were generated for this study. This is an essayistic article based on bibliographical sources.
