# Supplementary material for: Patterns of Reimperialization and Postimperial strategic cultures. The cases of Liberal Spain (1833-1868) and Post-Soviet Russia (1991-2025)
Source: Open Res Eur. 2026 Jul 22;5:266. Originally published 2025 Sep 2. [Version 2] doi: 10.12688/openreseurope.21137.2 (PMC13560817; doi:10.12688/openreseurope.21137.2)
Supplement: Peer review history

# Reviewer report: Reviewer response for version 2

**DOI:** 10.21956/openreseurope.26632.r78591

**Published:** 2026-08-27

**Authors:** Paul Flenley

**Affiliations:** 1 University of Portsmouth, Portsmouth, UK

I found this to be a very insightful and imaginative study. At first the two case studies may seem rather an odd selection both temporally and geographically.

 However, the comparison works extremely well not only in bringing out similarities in what are two non-standard areas of study in decolonization but also identifying differences which help to illustrate the specificity of the post-imperial transition in each case. Also the article comes at an opportune moment for the study of the Russo-Ukraine war.

 The phenomenon of former imperial centres trying to sustain influence over their former imperial spaces is well studied. However this comparative study adds to our understanding of how and why this occurs especially in two cases which are generally not the geographical topic of focus for decolonial studies. The article makes the key points that the collapse of empire in both these cases was such that it undermined the core of the empire itself in part because of the speed of the collapse but also because these two cases did not just **have **empires but **were** empires. The lack of a transition to a coherent nation state with a successful market and ideology led to a strategic culture which tried to link internal regeneration to the creation of post imperial spheres of influence i.e. re-imperialisation linked to internal reconstruction. The article shows how failures in the application of soft power to achieve this rapidly led to the use of hard power. The article contains a rich range of insights such as the way in which the excuse of protecting a diaspora or ensuring security enabled intervention in the former imperial space to be articulated as an act of responsibility. The article also points out how the international context e,g, NATO/EU enlargement and the perceived weakness of potential opposition e.g. US withdrawal from Afghanistan facilitated the shift to the use of hard power.

 The article makes very good integrated use of relevant theory such as historical institutionalism and strategic studies. It is also adept at showing how the use of concepts such as ‘levels of imperiality’ can enable us to pursue a more nuanced approach to the extent and nature of links between the core of a former empire and successor states. This helps us counter the accusation that the Soviet Union was not in fact an empire in the classic sense and allows us still to use the term the re-imperialization when analysing the Russo-Ukraine war.

 The article is aware of key differences between the two case studies, not least the fact that Russia has the advantage of geographical proximity to its successor states. It also provides a clear framework for identifying the transition processes of collapse to re imperialization but it makes clear that this is not an overall framework for the study of the phenomenon but a contribution to analysis which may or may not be applicable elsewhere especially where imperial collapse is sudden. Not all transitions from empire result in the use of hard power to re-imperialize or to maintain connections.

 In short this is a very clearly written and well-argued study of post imperial transition in two specific cases. The comparison is well structured and suitably detailed. It draws on a wide range of relevant literature. It is particularly helpful in understanding the current conflict in Ukraine and Russian policymaking.

Is the argument persuasive and supported by appropriate evidence?

Yes

Is the work clearly and cogently presented?

Yes

Is the topic of the essay discussed accurately in the context of the current literature?

Yes

Does the essay contribute to the cultural, historical, social understanding of the field?

Yes

Reviewer Expertise:

International Relations and Politics - especially Russian foreign policy, relations between NATO/the EU and Eastern Neighbourhood; Russian/Soviet history.

I confirm that I have read this submission and believe that I have an appropriate level of expertise to confirm that it is of an acceptable scientific standard.

# Reviewer report: Reviewer response for version 2

**DOI:** 10.21956/openreseurope.26632.r77827

**Published:** 2026-08-07

**Authors:** Yimovie Sakue-Collins

**Affiliations:** 1 University of Africa, Toru-Orua, Nigeria

The manuscript has improved considerably and adequately addresses the issues I raised earlier. I recommend it for indexing.

Is the argument persuasive and supported by appropriate evidence?

Partly

Is the work clearly and cogently presented?

Yes

Is the topic of the essay discussed accurately in the context of the current literature?

Yes

Reviewer Expertise:

NA

I confirm that I have read this submission and believe that I have an appropriate level of expertise to confirm that it is of an acceptable scientific standard.

# Reviewer report: Reviewer response for version 2

**DOI:** 10.21956/openreseurope.26632.r77826

**Published:** 2026-07-31

**Authors:** Marco Marsili

**Affiliations:** 1 ISCTE-University Institute of Lisbon Center for International Studies, Lisbon, Lisbon, Portugal

1. Constructive Assessment of the Revised Study

 The author has produced an exemplary revision that addresses the concerns raised in my original peer review report with remarkable thoroughness and scholarly care. The revised version is significantly stronger, more nuanced, and theoretically robust than its predecessor. The comprehensive response demonstrates serious engagement with the review process and a genuine commitment to improving the work's analytical depth and scholarly contribution.

**Key Improvements Noted:**

- The introduction has been substantially expanded to better justify the selection of post-Soviet Russia as a comparative case, explicitly acknowledging the historiographical debate on the imperial nature of the USSR while persuasively demonstrating why it can be treated as an imperial regime.
- The theoretical framework has been reorganized and enlarged, providing explicit definitions of empire, imperialism, reimperialization, levels of imperiality, and degrees of rupture. The relational definition of empire as "organized, institutionalized, and hierarchized multipolitical networks of power" provides a firm analytical foundation.
- The manuscript now systematically weaves the explanatory variables for divergent outcomes (internal consolidation of the core, stability of the periphery, international permissiveness) throughout the historical narrative, creating a more dynamic and dialectical comparison.
- The engagement with realist counterarguments is now explicit and sophisticated, positioning the reimperialization framework as a necessary complement to—rather than a replacement for—systemic explanations.
- The concept of "Americanism" is now defined explicitly upon introduction and contextualized within the historiography of Latin American political thought.
- The discussion of the political language of protection has been significantly expanded, demonstrating how the security of expatriate communities provides "the legal and moral vocabulary through which intervention could be presented as defensive rather than expansionist."
- The conclusion now presents the six-stage model explicitly as a heuristic framework rather than a deterministic theory, clarifying the broader applicability and limits of the concept of reimperialization.

**Theoretical Innovation:** The introduction of the concepts of "levels of imperiality" and "degrees of rupture" continues to provide a novel and valuable analytical framework, effectively moving beyond the traditional "colonial/postcolonial" binary.

**Rigorous Comparative Framework:** The case selection remains bold and unconventional but is now even more convincingly justified. The article successfully demonstrates that despite vast chronological and technological differences, the two cases exhibit profound structural parallels in their strategic rationales and sequential progression.

**Exceptional Interdisciplinary Synthesis:** The manuscript skillfully synthesizes theories and methodologies from historical sociology, strategic studies, and institutionalism, with the engagement with scholars like Doyle (metrocentric/pericentric/systemic analysis) and Motyl (temporality of imperial collapse) now more deeply integrated.

**Clarity and Structure:** The argument is logically structured, moving coherently from theoretical framework to historical narrative to comparative analysis. The writing is clear and engaging.

**2. Answers to Mandatory Reviewer Questions**

 2.1. Is the article scientifically sound in its current form?

**Yes.** The article is scientifically sound. The methodology is clearly outlined and appropriate for a comparative historical study. The revisions have strengthened the theoretical framework, addressed potential counterarguments, and refined the comparative analysis. The argument is logically constructed, well-referenced, and engages seriously with relevant theoretical literature from multiple disciplines. The conclusions are supported by the evidence and analysis presented.

 2.2. Are the interpretations and conclusions justified by the results?

**Yes.** The author carefully builds the case for the six-stage pattern of reimperialization through a detailed and parallel historical narrative of both cases. The comparative conclusions about the role of imperiality, strategic culture, and international context are insightful, well-founded, and logically derived from the preceding analysis. The distinction between analogous strategies and equivalent capacities is now clearly maintained throughout.

 2.3. Is the article presented in an intelligible fashion and written in standard English?

**Yes.** The article is well-written and intelligible. The prose is academic but clear. I note only one minor formatting inconsistency in the revised version (on page 8 of the revised manuscript, a small formatting issue appears in a reference to note "3¹") which does not detract from the overall clarity.

 2.4. Does the article meet the criteria of publication in Open Research Europe in terms of subject matter and academic quality?

**Yes, absolutely.** The article aligns perfectly with the journal's scope, promoting open, interdisciplinary, and high-impact research. Its subject matter is of significant contemporary relevance, and its academic quality—in terms of theoretical innovation, comparative rigor, and scholarly engagement—is excellent and meets a high standard of scholarship.

 2.5. Do you have any concerns regarding research ethics or plagiarism?

**No.** I have no concerns regarding research ethics or plagiarism. The article properly cites a wide range of sources, and its argument, while synthesizing existing ideas, is original in its comparative scope, theoretical framework, and specific conclusions.

 3. Aspects Not Assessed

 As the article is explicitly a theoretical and bibliographic essay, the standard assessment of original empirical data collection or laboratory methodologies does not apply. The data availability statement is accurate and appropriate for this article type.

 4. Additional Considerations

 The author has addressed the concerns raised in my original report with exemplary scholarly rigour. The revised manuscript is theoretically innovative, analytically robust, and empirically well-supported within the limits of its essayistic methodology. It makes a significant contribution to the interdisciplinary study of post-imperial conflicts and strategic cultures.

 The article's contribution extends beyond the specific cases of Spain and Russia. By demonstrating that both powers followed analogous sequences of post-imperial behaviour despite their vastly different geographical, technological, and geopolitical contexts, the author challenges linear and teleological views of decolonization. The recognition that empire does not simply end with political independence or territorial loss, but rather persists in institutional frameworks, strategic imaginations, and political calculations, is essential for understanding the resurgence of imperial logics in the twenty-first century.

**I consider this manuscript ready for final indexing in Open Research Europe.**

 5. Updated Approval Status

 The author has addressed the concerns raised in my original report thoroughly and effectively. I am pleased to upgrade my approval status from "Approved with Reservations" to **"Approved."**

Is the argument persuasive and supported by appropriate evidence?

Yes

Is the work clearly and cogently presented?

Yes

Is the topic of the essay discussed accurately in the context of the current literature?

Yes

Does the essay contribute to the cultural, historical, social understanding of the field?

Yes

Reviewer Expertise:

Global affairs, int. studies, int. relations, political science, contemporary history, human rights, int. law, int. humanitarian law

I confirm that I have read this submission and believe that I have an appropriate level of expertise to confirm that it is of an acceptable scientific standard.

# Reviewer report: Reviewer response for version 1

**DOI:** 10.21956/openreseurope.22865.r63412

**Published:** 2025-12-11

**Authors:** Yimovie Sakue-Collins

**Affiliations:** 1 University of Africa, Toru-Orua, Nigeria

I recommend this manuscript be indexed with minor revision(s).  

 The manuscript contains all the essential components of a strong contribution, theoretically and empirically. It is already a well-worked out manuscript. Case study of this type alongside theoretical contributions is invaluable in this era of proliferation of imperial ethos.  However, several clarifications would strengthen its core argument and enhance its theoretical impact. Most importantly, the introduction does not sufficiently situate Russia (whether it is pre-Soviet, Soviet, and post-Soviet phases) as a case study. Crucially, there is need for context, since the entire idea of reimperialisation (the core of the paper) rests on the understanding of Russia as an empire. Readers will want to understand why Russia is illustrative for the argument on reimperialisation; what makes it comparable or distinct from other imperial cases, and how this case advances the theoretical intervention. A brief, focused section addressing imperialism as an expansionist tendency of empire, before the discussion of the “levels of imperiality,” would help establish this foundation. Such framing would also position the Russian case in relation to the more familiar Spanish example and connect it to contemporary debates about Russia’s relationships with other postcolonial states. Conspicuously, the absence of this grounding is profound, and echoes through the manuscript. 

 On page 7, for instance, Russia is introduced through the statement that “the USSR did not undergo an orderly imperial dismantling that preserved the stability of its core,” which presumes an already-accepted classification of the USSR as an empire. The subsequent explanation, however, aligns more closely with the dissolution of a confederation. The concern here is that because the manuscript’s central concept – reimperialisation – rests on how “empire” is understood, the paper would benefit from a clearer operationalisation of empire in the context of the study. Is imperial status defined by territorial vastness, by the forcible annexation of constituent units, or by ideological cohesion such as the “binding force of communist ideology”? Given ‘ongoing debates’ over whether the USSR was – or still is – an empire, providing clarity here is essential before analysing its disintegration as an imperial collapse. This clarification is especially important because, unlike Spain, an uncontested and notorious imperial power with overseas possessions – Russia’s imperial status is historically and analytically complex. Establishing the grounds on which the USSR is considered an empire will help readers follow the subsequent argument about its disintegration and the application of the notion of reimperialisation.

 The manuscript also draws conceptual parallels between Spain and Russia, yet the mechanisms motivating their expansionist tendencies appear differently elaborated. In the Spanish case, the text convincingly shows how “[n]ationalism, militarism, and post-imperial” ambition interacted to regenerate imperial assertiveness. And this was supported with "[t]he re-expansion campaigns in Asia, the Caribbean, Morocco, and the Pacific articulated a discourse celebrating Spain's geopolitical regeneration" (p.11). In contrast, the Russian case is explained primarily through “anti-Westernism and irredentism,” which are presented as if directly equivalent to imperialism. Further clarification would help here: Is anti-Western sentiment inherently expansionist? Do irredentist claims necessarily amount to imperial ambition, especially when such claims are framed by some groups (and even states) as responses to past imperial fragmentation? Without addressing these gaps, “unequivocal” conclusions such as the one reached on page 8 (para 3) appear questionable.

 Relatedly, a typology which exclusively lists rising non-Western powers such as “Russia, China, Turkey, and Iran” (p.4) for theory development, would benefit from additional explanation. Without further context, it risks appearing “revisionist,” and some claims would be strengthened by nuance rather than assertion. For instance, while non-Western powers are sometimes positioned as revisionist or expansionist, it is equally well-documented that established Western states (e.g., the US, France, Britain) have frequently violated the principle of Westphalian sovereignty when it aligns with their interests. Acknowledging these complexities would help avoid over-generalisation. [Similarly, though beyond the scope of this manuscript – but nonetheless cited, this need for nuance also applies to the discussion of the People's Republic of China (PRC) and the Republic of China (ROC or Taiwan) relationship. While the PRC’s contemporary claims over Taiwan may be interpreted as ‘reimperialising’; China’s imperial claim over the island is also contested. The contemporary history of cross-strait relations is shaped by the unresolved Chinese Civil War, in which both the PRC and ROC claim sovereignty over the same territory. Thus, recognising this layered history will prevent the argument from appearing to apply a general rule too broadly.]

 On theory and theoretical development

 Additionally, the manuscript would benefit from a clearer and more fully developed theoretical grounding integrating the different perspectives highlighted. At present, perspectives such as “serial contextualism,” “structural studies of imperial cycles,” “strategic culture studies” and “historical institutionalism” are cited without sufficient explanation of their basic assumptions, how they connect – fit or complement each other, or relevance to the analysis. This weakens the theoretical section and contributes to some of the broader gaps in the argument. Given the manuscript’s ambition, the theoretical framework should be more explicit and robust, offering readers a clear set of conceptual tools with which to follow the analysis. Strengthening this section can be done straightforwardly by operationalising the key theories early in the manuscript and addressing four essential elements: (a) a synthesis of the context of the ‘theories’/perspectives; (b) the core assumptions and concepts (the latter is to some extent addressed); (c) the primary exponents and relevant variations; and (d) their specific application to the present study (partially addressed). Doing so will provide readers with a stronger frame of reference and reinforce the contribution the manuscript aims to make.

 Overall, the manuscript will be considerably strengthened by: (i) dedicating a brief focused section on imperialism, clarifying the criteria by which the USSR/Russia is framed as an empire; (ii) strengthening the section on theoretical framework (“levels of imperiality”) to allow for; (iii) more fully contextualising expansionist tendencies or identifying variables that link both cases; and (iv) addressing areas where nuance is needed to avoid over-generalisation. These revisions will help ensure that the argument for reimperialisation is analytically robust, theoretically compelling, and more general for postimperial conflict analysis.

Is the argument persuasive and supported by appropriate evidence?

Partly

Is the work clearly and cogently presented?

Yes

Is the topic of the essay discussed accurately in the context of the current literature?

Yes

Reviewer Expertise:

NA

I confirm that I have read this submission and believe that I have an appropriate level of expertise to confirm that it is of an acceptable scientific standard, however I have significant reservations, as outlined above.

# Response

**Published:** 2025-12-17

**Authors:** Rodrigo Escribano Roca

Dear Reviewer, I am very grateful for your careful, insightful, and generous evaluation of the manuscript. I sincerely appreciate the time and intellectual attention you have devoted to the paper, as well as the constructive and precise nature of your comments. Your observations are both intelligent and well-targeted, and they engage directly with the core conceptual and theoretical ambitions of the article. In particular, I find your remarks on the need to clarify the operationalisation of empire in the Russian/Soviet case, to strengthen the theoretical framework, and to introduce greater nuance in the discussion of expansionism and revisionism extremely helpful. These suggestions not only identify areas that require clarification but also point to concrete ways in which the manuscript’s analytical coherence and theoretical contribution can be significantly improved. I also appreciate your recognition of the manuscript’s overall contribution and potential, which is very encouraging. I will revise the article thoroughly in light of all your comments and will incorporate your suggestions carefully in a new version of the manuscript. I expect to submit this revised version, together with a detailed response to the review, in the near future. Thank you again for your thoughtful and constructive engagement with my work. Kind regards,

 Rodrigo Escribano Roca

# Reviewer report: Reviewer response for version 1

**DOI:** 10.21956/openreseurope.22865.r59808

**Published:** 2025-09-11

**Authors:** Marco Marsili

**Affiliations:** 1 ISCTE-University Institute of Lisbon Center for International Studies, Lisbon, Lisbon, Portugal

Constructive Assessment of the Study

 This article presents a highly ambitious and intellectually stimulating comparative analysis of post-imperial strategic cultures in Liberal Spain (1833-1868) and Post-Soviet Russia (1991-2025). The core argument—that both states, following abrupt imperial collapse, developed strategic cultures oriented toward reimperialization and followed a comparable six-stage pattern—is original, timely, and has significant potential to contribute to the fields of historical sociology, international relations, and strategic studies.

 Major Strengths:

- Theoretical Innovation: The introduction of the concepts of "levels of imperiality" and "degrees of rupture" provides a novel and valuable analytical framework. It effectively moves beyond the traditional "colonial/postcolonial" binary to offer a more nuanced tool for comparing the institutional and ideological legacy of empires within their former metropolitan cores.
- Rigorous Comparative Framework: The case selection is bold and unconventional but is convincingly justified. The article successfully demonstrates that despite vast chronological and technological differences, the two cases exhibit profound structural parallels in their strategic rationales and sequential progression. The application of a historical institutionalist approach to explain the path dependency of strategic cultures is particularly well-executed.
- Exceptional Interdisciplinary Synthesis: The manuscript skillfully synthesizes theories and methodologies from historical sociology, strategic studies, and institutionalism. The engagement with scholars like Doyle (metrocentric/pericentric/systemic analysis) and Motyl (temporality of imperial collapse) deepens the comparative analysis and provides a robust theoretical backbone.
- Clarity and Structure: The argument is logically structured, moving coherently from theoretical framework to historical narrative to comparative analysis. The writing is clear and engaging, making a complex argument accessible.

Recommendations for Improvement:

- Nuance the Parallels, Integrate the Differences: While the parallels are compelling, the analysis would be strengthened by a more systematic and earlier integration of the *variables that explain the different outcomes* (Spain's failure vs. Russia's persistent aggression). The excellent discussion of Motyl's framework on the conditions for successful irredentism appears late in the article; weaving these variables (internal consolidation of the core, stability of the periphery, international permissiveness) more explicitly throughout the historical narrative in sections 3.2 and 3.3 would create a more dynamic and dialectical comparison.
- Engage with Counterarguments: The article convincingly presents its case but could enhance its persuasiveness by preemptively addressing potential counterarguments. For instance, a paragraph could acknowledge and refute the alternative explanation that Russia's actions are primarily driven by classic great power realism rather than a distinct "post-imperial" logic. Explicitly stating why the "reimperialization" framework provides a superior explanation would be valuable.
- Source Base and Historiographical Engagement: The bibliography is extensive. To further elevate the manuscript, the author could, where possible, integrate more primary source evidence (e.g., deeper analysis of diplomatic dispatches, political speeches, or contemporary press) to complement the strong theoretical and secondary source synthesis, particularly for the Spanish case.
- Define Key Concepts upon First Use: The concept of "Americanism" as a strategic culture binding the South American republics is fascinating and crucial to the argument. It would be beneficial to define this term more explicitly when it is first introduced, anchoring it in the relevant historiography of Latin American political thought.

Aspects Not Assessed:

 As the article is explicitly a theoretical and bibliographic essay, the standard assessment of original empirical data collection or laboratory methodologies does not apply. The data availability statement is accurate and appropriate for this article type.

 1. Constructive Assessment of the Study

 This article presents a highly ambitious and intellectually stimulating comparative analysis of post-imperial strategic cultures in Liberal Spain (1833-1868) and Post-Soviet Russia (1991-2025). The core argument—that both states, following abrupt imperial collapse, developed strategic cultures oriented toward reimperialization and followed a comparable six-stage pattern—is original, timely, and has significant potential to contribute to the fields of historical sociology, international relations, and strategic studies.

 Major Strengths:

- Theoretical Innovation: The introduction of the concepts of "levels of imperiality" and "degrees of rupture" provides a novel and valuable analytical framework. It effectively moves beyond the traditional "colonial/postcolonial" binary to offer a more nuanced tool for comparing the institutional and ideological legacy of empires within their former metropolitan cores.
- Rigorous Comparative Framework: The case selection is bold and unconventional but is convincingly justified. The article successfully demonstrates that despite vast chronological and technological differences, the two cases exhibit profound structural parallels in their strategic rationales and sequential progression. The application of a historical institutionalist approach to explain the path dependency of strategic cultures is particularly well-executed.
- Exceptional Interdisciplinary Synthesis: The manuscript skillfully synthesizes theories and methodologies from historical sociology, strategic studies, and institutionalism. The engagement with scholars like Doyle (metrocentric/pericentric/systemic analysis) and Motyl (temporality of imperial collapse) deepens the comparative analysis and provides a robust theoretical backbone.
- Clarity and Structure: The argument is logically structured, moving coherently from theoretical framework to historical narrative to comparative analysis. The writing is clear and engaging, making a complex argument accessible.

Recommendations for Improvement:

- Nuance the Parallels, Integrate the Differences: While the parallels are compelling, the analysis would be strengthened by a more systematic and earlier integration of the *variables that explain the different outcomes* (Spain's failure vs. Russia's persistent aggression). The excellent discussion of Motyl's framework on the conditions for successful irredentism appears late in the article; weaving these variables (internal consolidation of the core, stability of the periphery, international permissiveness) more explicitly throughout the historical narrative in sections 3.2 and 3.3 would create a more dynamic and dialectical comparison.
- Engage with Counterarguments: The article convincingly presents its case but could enhance its persuasiveness by preemptively addressing potential counterarguments. For instance, a paragraph could acknowledge and refute the alternative explanation that Russia's actions are primarily driven by classic great power realism rather than a distinct "post-imperial" logic. Explicitly stating why the "reimperialization" framework provides a superior explanation would be valuable.
- Source Base and Historiographical Engagement: The bibliography is extensive. To further elevate the manuscript, the author could, where possible, integrate more primary source evidence (e.g., deeper analysis of diplomatic dispatches, political speeches, or contemporary press) to complement the strong theoretical and secondary source synthesis, particularly for the Spanish case.
- Define Key Concepts upon First Use: The concept of "Americanism" as a strategic culture binding the South American republics is fascinating and crucial to the argument. It would be beneficial to define this term more explicitly when it is first introduced, anchoring it in the relevant historiography of Latin American political thought.

Aspects Not Assessed:

 As the article is explicitly a theoretical and bibliographic essay, the standard assessment of original empirical data collection or laboratory methodologies does not apply. The data availability statement is accurate and appropriate for this article type.

 3. Additional Considerations for the Author

 The suggestion to consider integrating insights from the *Review of Human Rights *( https://reviewhumanrights.com/index.php/RHR/article/view/282) is excellent. If the source proves relevant, it could add a valuable layer to your analysis by:

- Providing a critical framework for analyzing how narratives of human rights and protection are instrumentalized by post-imperial powers to legitimize interventionism (e.g., protection of diasporas).
- Strengthening the interdisciplinary nature of the paper by engaging with legal and philosophical critiques of universalism, which align with your discussion of challenges to the Westphalian/Wilsonian order.

Is the argument persuasive and supported by appropriate evidence?

Yes

Is the work clearly and cogently presented?

Yes

Is the topic of the essay discussed accurately in the context of the current literature?

Yes

Does the essay contribute to the cultural, historical, social understanding of the field?

Yes

Reviewer Expertise:

Global affairs, int. studies, int. relations, political science, contemporary history, human rights, int. law, int. humanitarian law

I confirm that I have read this submission and believe that I have an appropriate level of expertise to confirm that it is of an acceptable scientific standard, however I have significant reservations, as outlined above.
